# Clinical and biological features associated to bipolar disorder with comorbid migraine: results from the FACE-BD cohort

**DOI:** 10.1192/j.eurpsy.2023.839

**Published:** 2023-07-19

**Authors:** M.-C. Patoz, O. Godin, X. Moisset, J. Chabert, K. M’Bailara, B. Etain, R. Belzeaux, C. Dubertret, E. Haffen, R. Schwan, P. Roux, M. Polosan, V. Aubin, M. Leboyer, P. Courtet, E. Olie, P.-M. Llorca, L. Samalin

**Affiliations:** 1 CHU Clermont-Ferrand, Université Clermont Auvergne, Institut Pascal, clermont ferrand; 2 fondation fondamental, créteil; 3 Université Clermont Auvergne, CHU de Clermont-Ferrand, Inserm, Neuro-Dol, clermont ferrand; 4 LabPsy, University of Bordeaux, EA 4139; 5 Department of Clinical and Academic Psychiatry, Charles-Perrens Hospital, Bordeaux; 6 AP-HP, GHU Paris Nord, DMU Neurosciences, Hôpital Fernand Widal; 7 INSERM UMRS 1144-Université de Paris, Paris; 8 Pôle de Psychiatrie, Assistance Publique Hôpitaux de Marseille; 9 INT-UMR 7289, CNRS Aix-Marseille Université, Marseille; 10 Department of Psychiatry, University of Paris, AP-HP, Louis Mourier Hospital, INSERM UMR 1266 PARIS, Colombes; 11 Service de Psychiatrie de l’Adulte, CIC-1431 INSERM, CHU de Besançon, Laboratoire de Neurosciences, Université de Franche-Comté, UBFC, Besançon; 12 Université de Lorraine, Centre Psychothérapique de Nancy, Pôle Hospitalo-Universitaire de Psychiatrie d’Adultes du Grand Nancy, INSERM U1254, Nancy; 13 Centre Hospitalier de Versailles, Le Chesnay, EA 4047 HANDIReSP, UFR des Sciences de la Santé Simone Veil, Université Versailles Saint-Quentin-en-Yvelines, Versailles, France and Université Paris-Saclay, UVSQ, Inserm, CESP, Equipe “PsyDev”, Villejuif; 14 Université Grenoble Alpes, Inserm U1216, Grenoble Institut de Neurosciences, CHU de Grenoble, Grenoble; 15 Pôle de Psychiatrie, Centre Hospitalier Princesse Grace, Monaco; 16 Université Paris Est Creteil (UPEC), AP-HP, Hôpitaux Universitaires «H. Mondor», DMU IMPACT, INSERM, IMRB, Translational Neuropsychiatry, Créteil; 17 Institute of Functional Genomics, University of Montpellier, CNRS, INSERM; 18 Department of Emergency Psychiatry and Post-Acute Care, CHU Montpellier, Montpellier; 19 Department of Emergency Psychiatry and Post-Acute Care, CHU Montpellier, Montpellier; 20 CHU Clermont-Ferrand, Université Clermont Auvergne, Institut Pascal, Clermont-Ferrand, France

## Abstract

**Introduction:**

Migraine and bipolar disorder (BD) are two chronic and recurrent disorders with a major impact on patient’s quality of life. It is now well known that affective disorders and migraine are often comorbid (Leo *et al.* Scand J Pain. 2016; 11:136-145). Starting from these observations, we can hypothesis that BD patients with comorbid migraine might have specifical clinical and biological features.

**Objectives:**

The aim of this study was to estimate the prevalence of migraine in a cohort of French BD patients; determine sociodemographic, clinical, and biological features associated BD-migraine comorbidity.

**Methods:**

4348 BD patients from the FACE-BD cohort were included from 2009 to 2022. Sociodemographic and clinical characteristics, lifestyle information, and data on antipsychotic treatment and comorbidities were collected, and a blood sample was drawn. The Structured Clinical Interview for DSM-IV Axis I Disorders was used to confirm the diagnosis of BD. Migraine diagnosis was established according to a clinician-assessed questionnaire.

**Results:**

20.1% of individuals with BD had comorbid migraine. Half of these patients received treatment for migraine. Multivariate logistic regression model showed that risk of migraine in women was nearly twice that in men (OR = 1.758; 95% CI, 1.345-2.298). Anxiety disorder, sleep disturbances and childhood trauma were also associated with an increased risk of migraine comorbidity. Patients receiving antipsychotic treatment had less risk of developing migraine than those not receiving those treatment (OR 0.716, 95% CI, 0.554-0.925), independent of other potential confounders.

**Conclusions:**

The prevalence of migraine in our cohort was lower than those previously reported in other studies. This result might suggest an overestimation of migraine diagnosis in BD patients population studies. However, BD-migraine comorbidity could constitute a subphenotype of bipolar disorder requiring specific treatments.

**Disclosure of Interest:**

None Declared

